# A hitchhiker's guide to modern, practical cyanobacterial taxonomy

**DOI:** 10.1111/jpy.70102

**Published:** 2025-11-12

**Authors:** Petr Dvořák, Svatopluk Skoupý, Aleksandar Stanojković, Jeffrey R. Johansen, Chelsea Villanueva, Patrick Jung, Laura Briegel‐Williams, H. Dail Laughinghouse, Forrest W. Lefler, David E. Berthold, Jan Kaštovský, Anne C. Hurley, Dale A. Casamatta

**Affiliations:** ^1^ Department of Botany, Faculty of Science Palacký University Olomouc Olomouc Czech Republic; ^2^ National Marine Fisheries Research Institute Department of Fisheries Oceanography and Microbial Ecology Gdynia Poland; ^3^ Department of Biology John Carroll University University Heights Ohio USA; ^4^ Department of Botany, Faculty of Science University of South Bohemia České Budějovice Czech Republic; ^5^ Department of Biological, Geological, & Environmental Sciences Cleveland State University Cleveland Ohio USA; ^6^ Extreme Cryptogam Ecology Lab University of Applied Sciences Kaiserslautern Kaiserslautern Germany; ^7^ Fort Lauderdale Research and Education Center, IFAS University of Florida Davie Florida USA; ^8^ Department of Biology University of North Florida Jacksonville Florida USA

**Keywords:** bioinformatics, cyanobacteria, ITS, systematics, taxonomy

## Abstract

There has been an explosion of new Cyanobacterial taxa described within the last two decades. Cyanobacteria exhibit incredible ecological versatility and morphological variability, and thousands of species have already been described using “traditional” approaches (e.g., morphological features). However, DNA sequencing and other molecular tools have provided extensive evidence that the diversity of cyanobacteria is not necessarily congruent with morphology, as many morphological genera (e.g., *Phormidium*, *Leptolyngbya,* and *Nostoc*) are polyphyletic, and species within the genera are often morphologically indistinguishable, thus cryptic. Further confounding systematic assessments, newly erected taxa are often based on a single strain with one or two 16S rRNA gene sequences, may have incomplete formal descriptions, and lack indication of the employed species concepts. Here we have proposed a set of guidelines for cyanobacterial taxonomists. We have focused on the whole process of erecting new taxa: sampling, sequencing (including genomes), phylogenetic inference, phenotype characterization, species concepts, formal descriptions, and codes of nomenclature. Our hope is that these guidelines will help with the laborious but ever‐rewarding task of identifying and describing the taxa within the world of cyanobacteria.

**Abbreviations**
AAI: average amino‐acid identity
ANI: average nucleotide identity
BLAST: Basic Local Alignment Search Tool
CIMS: Cyanobacterial ITS Motif Slicer
GPS: global positioning system
ICN: International Code of Nomenclature of Plants, Algae, and Fungi
ICNP: International Code of Nomenclature of Prokaryotes
ICSP: International Committee on Systematics of Prokaryotes
IJSEM: International Journal of Systematic and Evolutionary Microbiology
IPNI: International Plant Names Index
ITS: internal transcribed spacer
MSA: multiple sequence alignment
NCBI: National Center for Biotechnology Information
PCR: polymerase chain reaction
SeqCode: Code of Nomenclature of Prokaryotes Described from Sequence Data
SRA: Sequence Read Archive
WGA: whole‐genome amplification
WGS: whole‐genome sequencing

“Don't Panic.”—Douglas Adams, The Hitchhiker's Guide to the Galaxy

## INTRODUCTION

Hundreds of new cyanobacterial taxa have been proposed in the last 2 decades (reviewed in Strunecký et al., 2023). The beginning of this great boom coincided with the promulgation and accessibility of molecular methods (i.e., easy, inexpensive Sanger sequencing). Early researchers employing these methods suggested that some morphological features evolved in parallel or convergently many times (e.g., Turner, 1997; Wilmotte, 1994). In terms of taxonomy, this led to an atomization of the morphologically defined genera, which had up to that point been the de facto method for articulating biodiversity. For instance, Dvořák et al. (2014) reconstructed a phylogeny of *Synechococcus* spp. using 16S rRNA gene sequences and determined that this cosmopolitan genus in fact encompassed 12 separate lineages, which although nearly morphologically identical, were genetically distinct enough to be classified as separate genera (e.g., *Neosynechococcus*, Dvorák et al., 2014; *Thermostichus*, Komárek et al., 2020).

Both morphological crypsis and phenotypic plasticity have led to taxonomic confusion (e.g., Berthold et al., 2021; Casamatta et al., 2003; Johansen & Casamatta, 2005; Villanueva et al., 2018). Thus, morphological criteria are often not sensitive enough to recognize species boundaries. For instance, Osorio Santos et al. (2014) proposed seven species of *Oculatella* based on 16S rRNA gene and 16S–23S ITS rRNA region sequences, including some that were impossible to separate simply using morphology. Even more morphologically complex genera may be rife with cryptic taxa. For example, *Compactonostoc*, *Minunostoc*, *Purpureonostoc*, *Desmonostoc, Halotia, Komarekiella, Desikacharya*, *Aliinostoc*, and *Pseudoaliinostoc* have all recently been split from *Nostoc* (Cai et al., 2019; Cai & Li, 2020; Hrouzek et al., 2013; Genuário et al., 2015; Hentschke et al., 2017; Saraf et al., 2019; Bagchi et al., 2017; Lee et al., 2021). To account for such difficulties in selecting morphological characters, a more holistic approach (akin to the approach known as MAAT, or molecular‐assisted alpha taxonomy to our macroalgal colleagues) has been advocated, one that employs genetic data in combination with morphological and ecological characteristics (the total evidence or polyphasic approach sensu Johansen & Casamatta, 2005).

In the past, it was hoped that a single molecular marker would be sufficient for articulating cyanobacterial systematics (e.g., the 16S rRNA gene). However, evidence has mounted that 16S rRNA gene sequences alone might not be sensitive enough to define species boundaries, so it has become increasingly common to employ ITS rRNA region structures as well (please refer to recommendation 6 below). Although initially effective for defining species in the past, current application of the ITS rRNA region(s) is in flux, and species boundaries may change. For example, many cyanobacteria possess multiple operonic variants of this region, and Villanueva et al. (2024) note that issues of homologous versus paralogous sequences need to be addressed. Furthermore, sampling and articulating the whole genome at the population level may be significantly more discriminating (e.g., Antonaru et al., 2023; Skoupý et al., 2024) than single‐locus approaches. For example, *Laspinema* was recently split from the polyphyletic genus *Phormidium* (Heidari et al., 2018). When *Laspinema* was sampled from a small puddle in the Czech Republic, eight strains were obtained, and their genomes sequenced. Although the 16S rRNA gene sequences and morphology were almost identical, analyses of whole genomes and gene flow revealed two distinct, deeply divergent clades (Dvořák et al., 2024; Stanojković et al., 2022). Analyses of gene composition suggested a difference at a micro‐niche level between the two species. Similar patterns have been observed in planktic taxa such as *Microcystis* and *Prochlorococcus* (Kashtan et al., 2014; Pérez‐Carrascal et al., 2019), and thus it is likely that such cryptic microdiversity will be observed in other species.

**FIGURE 1:**
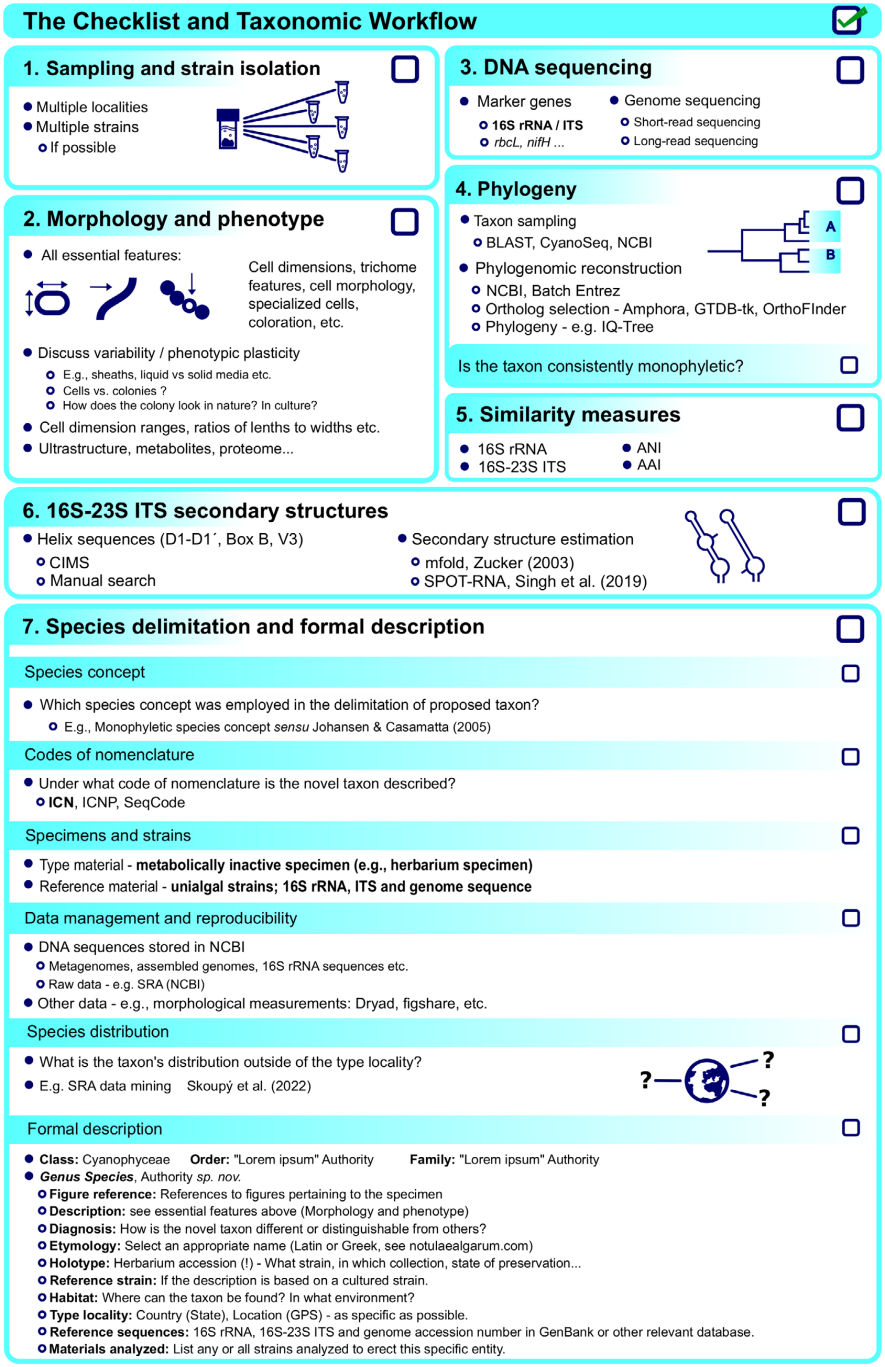
A checklist and the taxonomic workflow of recommendations that are presented in this paper. Each nomenclatural code has specific requirements which must be followed as well. These are the recommendations of the authors and not necessarily reflective of the current requirements of the Codes of Nomenclature.

How should one proceed in this evolving process of cyanobacterial systematics? After all, the goal of taxonomy is to name and articulate the biodiversity of the natural world in a practical manner (e.g., a pragmatic species concept). Thus, we propose the following seven recommendations and checklist (Figure 1), which we hope will help researchers with the difficult and much needed task of taxonomic articulation. These suggestions are designed mainly for novices and reviewers, but we hope that such a comprehensive review will be of help also to the more experienced researchers. Please note that much of the following material represents our collective recommendations for those articulating novel cyanobacterial diversity. Although not absolutely necessary under the nomenclatural codes (see Codes of Nomenclature section), information is provided that will help strengthen arguments that researchers are fully and accurately (to the greatest extent possible) describing the novel taxon. Also, we encourage reviewers of cyanobacterial taxonomy papers to keep these recommendations in mind.

## SAMPLING AND STRAIN ISOLATION

### Sampling—site information

It is helpful and prudent to give the geographical location of the sampling site, along with a description of site characteristics, as this provides valuable information about the taxon's ecology and distribution. As such, GPS coordinates and general climatic data for the sampling locality should be provided, in addition to relevant data regarding the particular sampling site. For samples from aquatic habitats, this may include water chemistry, flow velocity (for lotic systems), depth of occurrence (for lentic samples), substrate type (if material was sampled from submerged substrates such as rocks, aquatic plants, corals, etc.), and site abundance (e.g., is the taxon common or rare? Is it prevalent year‐round or restricted to certain seasons? etc.). For subaerial or terrestrial habitats, relevant information may include substrate type (e.g., soil, rock, plant surfaces, concrete walls or other man‐made structures, etc.), humidity and moisture (e.g., if material was sampled from wet rocks, damp soil, walls in a humid greenhouse, etc.), and sun exposure (e.g., unshaded/full exposure, partially or fully shaded by vegetation or man‐made structures). Cyanobacteria are ubiquitous, so we urge researchers to be thorough, regardless of from where the strains were isolated (e.g., epizooic, endophytic, etc.), and, if applicable, to give information on the host organism in terms of a symbiosis (e.g., the mycobiont of a lichen, plant, fern, etc.). We recommend that site data be provided directly in the text and not in reference to earlier publications. Images of the sampling sites are also welcome. They can be included in the main text or as supplemental material.

### Sampling—intraspecies diversity

To articulate intraspecies diversity, it is crucial to isolate multiple cultures from the investigated taxon from several localities (when possible) and to obtain several isolates from each (Barbosa et al., 2021; Berthold et al., 2021; Moretto et al., 2025; Pietrasiak et al., 2019, 2021; Stanojković et al., 2024). The largest genomic sampling effort for cyanobacterial populations was performed in the genus *Microcoleus* (genome sequences from 202 cultured cultures and eight metagenomes from herbarium specimens), revealing a continuum of barriers to gene flow coupled with genomic and morphological divergence (Skoupý et al., 2024; Stanojković et al., 2024). The species units ranged from morphologically distinguishable (with deep divergence and limited gene flow) to the cryptic (with relatively low divergence and frequent gene flow). The employment of a few conservative markers, coupled with only a few strains, may not be sufficient for revealing stages of speciation nor thus species delimitation. This emphasizes the importance of population sampling, genome sequencing, and understanding of the forces driving speciation for precise species delimitation (Dvořák et al., 2023). *Nota bene*: We employ the word “isolate” throughout this manuscript to describe the process of obtaining uni‐algal strains of cyanobacteria. We recognize that other fields (e.g., more traditional, heterotrophic bacterial systematists) may have a differing employment of this word, but in our context, it is merely that. Cyanobacteria are notoriously difficult to obtain as axenic strains for a variety of reasons (e.g., Lu et al., 2025; Morris et al., 2008), and as such, it is not typically practical to do so.

## PHENOTYPE ANALYSES

### Morphological observations

Although cyanobacterial taxonomy may be replete with cryptic species, many morphological traits are still useful for identification (e.g., Dvořák, Jahodářová, et al., 2015; Dvořák, Poulíčková, et al., 2015; Dvořák et al., 2023; Komárek et al., 2014; Komárek & Anagnostidis, 1998, 2005).

Words are funny: Much of their meaning is derived from context, which changes over time. Thus, formal descriptions should try to avoid subjective expressions such as “somewhat,” “more or less,” “slightly,” etc. These expressions are difficult to define and provide little guidance for identification. Although binary choices do not always represent the variety of morphology seen in nature, subjective expressions should be substituted with concrete terminology that accurately describes indeterminate morphology (e.g., “constricted at cross walls at an angle of X” instead of “slightly constricted at cross walls”). Formal descriptions should contain ranges of cell dimensions (and articulation of how many cells were measured in the Materials and Methods section), means, and standard deviations. Sheath/mucilage characteristics, color, nature of the thallus (both on the micro‐ and macroscopic levels), type of cell division, arrangement of thylakoids (if possible), presence or absence of aerotopes, production and patterns of specialized cells (e.g., nanocytes, baeocytes, akinetes, and heterocytes), colony shape, arrangement of cells in colonies (e.g., radially arranged, irregularly arranged, arranged in a single plane, etc.), filament polarity (heteropolar vs. isopolar), symmetry of filaments (with respect to heterocyte/akinete development), movement/motility, branching patterns, shape of terminal cells, and presence or absence of calyptra should all be assessed as appropriate. Special emphasis should be given on complex life cycles; all morphological stages should be assessed and characterized (e.g., vital in some taxa such as *Komarekiella* and *Cyanocohniella*). For heterocytous taxa, heterocyte shape, size, number, and placement are all significant for taxonomy, so attempt to provide as clear articulation as possible. When comparing several species and strains, the difference should be statistically analyzed using appropriate tests (e.g., Dvořák et al., 2024; Skoupý et al., 2024), ideally following common garden experiments under identical growth conditions and at similar life‐cycle stages (e.g., Reháková et al., 2007). Importantly, authors should clearly indicate if the morphological observations and measurements come from environmental samples or from strains under culture, as some commonly employed diagnostic features (e.g., sheaths, presence/absence of heterocytes, presence of hyaline hairs, etc.) may be culture dependent (Casamatta & Vis, 2004; Berrendero‐Gómez et al., 2016; Pokorný et al., 2023; Kaštovský, 2024).

Images are essential for unambiguously illustrating the morphological features of the investigated taxon. When preparing figures, either as micrographs or line drawings, care should be taken to display as wide a range of morphological variability as possible and as many of the assessed morphological features (see above) as possible. Because many features may change under culture conditions, images from both natural and cultured conditions should be given when possible. Consider both high and low magnification, with scale bars present. For taxa with complex three‐dimensionality (e.g., some of the mucilage‐encased colonial coccoids), drawings may be more feasible to illustrate features.

### Additional phenotypic traits

Besides the morphological traits, other phenotypic features may be investigated (e.g., the composition of fatty acids, secondary metabolites, photosynthetic pigments, diazotrophy, and cyanotoxins). Note that these traits, like morphological features, may be subject to convergent evolution, and therefore, their taxonomic value could be limited. For instance, chlorophyll *f*, a key pigment for far‐red light acclimation, has evolved in at least seven cyanobacterial lineages independently (Gisriel et al., 2023), and microcystins have been identified in six lineages across the Cyanobacterial phylum (reviewed in Dittmann et al., 2012).

## DNA SEQUENCING

### 16S rRNA gene and ITS rRNA region sequencing

The most common molecular markers used by cyanobacteriologists are the 16S rRNA gene and the 16S–23S ITS rRNA region. Although some researchers also employ other markers (e.g., the *rbc*L or *nif*H gene), fewer of these genes are in GenBank, thus hampering phylogenetic reconstruction. We recommend 16S rRNA gene and ITS rRNA region sequencing as the first step in the characterization of strains. We also advocate the employment of the primer set P1 (Wilmotte et al., 1993) and P2 (Nübel et al., 1997) as summarized in Boyer et al. (2001). Although this primer set does not sequence the whole 16S rRNA gene, it is very effective and reproducible and provides a long sequence in a single polymerase chain reaction (PCR) reaction, including the ITS rRNA region (ca. 1400–1700 bp). To obtain the complete 16S rRNA gene sequence, employ the cyanobacterial specific primer sets of Nübel et al. (1997). Additional primer sets can be located in Wilmotte et al. (2017).

### A note of caution

Many cyanobacteria contain multiple operonic variants, which may differ by several percentage points in sequence similarity (e.g., Johansen et al., 2017; Villanueva et al., 2024). Genomes of cyanobacteria with multiple rRNA operons may include combinations of paralogs and orthologs, with variable degrees of divergence (Saraf et al., 2024; Villanueva et al., 2024). Although there are several approaches in phylogenomics that allow the processing of paralogs (Li et al., 2003; orthoMCL etc.), it is much safer to use orthologs to impede the statistical noise in the phylogenetic inference. In order to circumvent these issues, researchers should contemplate employing cloning techniques when dealing with lineages in cases wherein this will likely be an issue (e.g., the *Nostocales*). Alternatively, long‐read genome sequencing allows assembly at the chromosome level and precise annotation of the ribosomal operon copies.

### Genome sequencing

Over the last decade, the price per sequenced base has dramatically decreased: Today, a draft genome of a cyanobacterium can be sequenced for <100 USD per strain using short‐read sequencing technologies (i.e., Illumina), and the full genome can be assembled for several hundred USD per strain using third‐generation sequencing platforms (e.g., Nanopore, PacBio). Whole genome sequencing (WGS) provides a rich source of information about the strain's evolution, physiology, and role in the environment. *We strongly recommend obtaining high‐quality draft or complete genome sequences for every proposed species*.

The short‐read sequencing technologies such as Illumina require the same DNA extraction protocol as for PCR amplifications (e.g., Moretto et al., 2025; Stanojković et al., 2022, 2024). The DNA library preparation protocol can be located, for example, in Modi et al. (2021), but commercial service may be cheaper and more suitable for less experienced users. The assembly of the genome comes with a high number of contigs and scaffolds because the short reads cannot bridge highly complex and repetitive regions. This issue can be ameliorated using third‐generation long‐read sequencing. The most popular technologies are PacBio (https://www.pacb.com/) and Oxford Nanopore (https://nanoporetech.com/). There is not enough space here to discuss the pros and cons of both technologies, but they are summarized in Lang et al. (2020). In short, PacBio is slightly more accurate, but Oxford Nanopore can sequence longer fragments of DNA (e.g., Cook et al., 2024). Third‐generation sequencing techniques require high molecular weight DNA with high levels of purity. Cyanobacterial cell walls, sheaths, and envelopes may cause issues during the high molecular weight DNA extraction (Hept & Greene, 2023).

The real challenge begins when you receive the raw sequencing data. For a typical pipeline for short‐read Illumina data, see Stanojković et al. (2024) and the related GitHub repository (https://github.com/dvorikus/Microcoleus‐population‐genomics). First, sequences need to be trimmed based on quality, followed by the removal of adapters and barcodes, and paired‐end reads need to be matched. Second, the filtered reads are assembled using one of many assembly pipelines. Bacteriologists frequently employ Spades (Bankevich et al., 2012), but there are many other popular assemblers (Segerman, 2020). Note that the third‐generation sequencing data require modified assembly protocols (see Cook et al., 2024 for the assembler benchmarking). Third, the assembled genomes often contain heterotrophic bacterial contaminants. We caution against simply using BLAST (https://blast.ncbi.nlm.nih.gov/Blast.cgi) because the BLAST would be time‐consuming (you would have to download the nucleotide database from NCBI and construct the library locally) and may not be precise enough. Cyanobacteria possess extremely large pangenomes (e.g., 133k genes in the genus *Microcoleus*; Stanojković et al., 2024); therefore, many contigs may be missed due to the incomplete database. We recommend using one or more binning pipelines instead, which evaluate the assembled contigs or scaffolds based on the tetranucleotide diversity, coverage, and identified core genes. Since the binning is developed for metagenomic sequencing, it may require manual corrections (see Nissen et al., 2021 for binning pipeline comparisons). Lastly, the final assembly is annotated using pipelines such as RAST (Aziz et al., 2008) or Prokka (Seemann, 2014).

### Single cell/colony/filament PCR, whole‐genome amplifications

Although many cyanobacterial species can be isolated into cultures using previously developed techniques (Dvořák et al., 2018), there are still many species that remain uncultured (e.g., Kilgore et al., 2018; Pokorný et al., 2023). Metagenomic sequencing techniques can circumvent this limitation, and several techniques have been developed to study genes and genomes from a single cell/filament, as well as their morphology. Single‐filament PCR requires a single cell, filament, or colony isolated directly into the PCR tube using a micropipette (see the following papers for developed methods: Boyer et al., 2001; Hašler et al., 2014; Mareš et al., 2019; Jung et al., 2024). High‐throughput approaches employ flow cytometry to sort cells into tubes or wells (Kashtan et al., 2014), where DNA is then directly extracted using freeze–thaw cycles and amplified using proofreading polymerases (Hašler et al., 2014; Mareš et al., 2019). Alternatively, the extracted DNA can be amplified using whole‐genome amplification (WGA) followed by regular PCR (e.g., Pokorný et al., 2023). The morphology of the cell/filament/colony can be evaluated under the microscope before DNA extraction, thus providing morphological data that can be connected with the DNA sequence data. It should be noted that isolated strains are preferred for species descriptions where possible because strains can be stored in the culture collection and used for further analyses in the future.

### Sequencing herbarium specimens

Whole genomes of centuries‐old herbarium specimens, including type specimens, can be sequenced (Skoupý et al., 2024; Stanojković et al., 2024). The protocols for low input DNA sequencing are available (Jungblut et al., 2021).

## PHYLOGENETIC AND PHYLOGENOMIC INFERENCE

The main point of a taxonomic proposal is to erect and describe novel biodiversity. To do so, scientists employ phylogenetic hypotheses (i.e., phylogenetic trees). The first and most crucial step of a phylogenetic analysis is taxon sampling (i.e., the selection of taxa for the analysis). Depending on the level of the taxonomic hypothesis, the most logistically feasible comprehensive data set needs to be obtained. To start, authors usually gather genome or gene sequences of their strains and search for additional data in GenBank (https://www.ncbi.nlm.nih.gov/) using BLAST (https://blast.ncbi.nlm.nih.gov/Blast.cgi) or keyword searches alongside relevant published works to identify closely related sequences (i.e., the most similar sequences in the GenBank database, as determined by BLAST search) and use keyword searches to gather sequences from other taxa of interest (e.g., sequences from the type species of a particular genus, sequences from representatives of a particular Order, etc.). Curated databases, such as CyanoSeq, may also be excellent starting points. CyanoSeq is the most up‐to‐date curated database of cyanobacterial 16S rRNA gene sequences for which complete data sets can be easily downloaded (Lefler et al., 2023). It should be mentioned that all data sets have to be manually proofed, since *many* erroneous taxonomic assignments (e.g., incorrectly identified strains) and sequences are stored in GenBank (Dvořák et al., 2018). Another great source of data is the Genome Taxonomy Database (https://gtdb.ecogenomic.org/), which allows users to browse and download genomes. Moreover, it provides a separate toolkit, GTDB‐tk (Parks et al., 2022), to identify studied genomes within its taxonomic framework.

Although 16S rRNA genes and ITS rRNA region sequences are directly aligned, whole‐genome data must be processed before the alignment and phylogeny by ortholog selection (ideally single‐copy orthologs). There are two possibilities: (1) to identify ubiquitous core single‐copy genes using tools such as Amphora (Wu & Eisen, 2008), GtoTree (Lee, 2019), or GTDB‐tk (Parks et al., 2022) and (2) to search all single‐copy orthologs using pipelines such as OrthoFinder (Emms & Kelly, 2019). The second approach can be beneficial, especially when studying relationships between populations or several species within a genus because it will identify thousands of genes. Thus, the resulting phylogeny has much higher statistical relevance.

Modern computers are powerful enough, and algorithms sophisticated enough, that maximum likelihood (ML) phylogenies of thousands of taxa can be estimated in a matter of hours (e.g., IQ‐TREE, Minh et al., 2020; RaxML‐NG, Kozlov et al., 2019). Thus, taxonomic studies focused on relationships higher than genera can contain representatives from the whole sequence space of the Cyanobacteria. Ideally, each taxonomic proposition should contain a phylogenetic tree with representatives from all orders of cyanobacteria to test if your studied *group is monophyletic* among Cyanobacteria. If proposing a new genus, the paper should include all known and available sequenced genera (specifically, the sequences of the type species from those genera) within the family. This also means that inclusion of the generitype is essential. In the case of a new species proposal, all species from the investigated genus must be sampled in addition to all related genera from the studied family (or even better, order, since greater representation should putatively lead to better phylogenetic resolution; see Lefler et al., 2023 for reference database). Of course, these suggestions may be modified due to a paucity of previously published sequences (e.g., there are relatively few coccoid sequences available for some lineages) or out‐of‐date taxonomy in databases, etc. Further, not all data sets are equally populated: 16S rRNA gene databases are significantly more extensive than databases for other genes or genomes. Thus, the taxon sampling needs to be adjusted according to the studied taxa and available data. Kapli et al. (2020) comprehensively reviewed the steps to phylogenetic reconstructions; it is beyond the scope of this paper to discuss technical and methodological considerations of the multiple sequence alignment and phylogenetic reconstruction.

## SEQUENCE SIMILARITY MEASURES

Researchers have long sought a single genetic similarity threshold that can serve as a universal solution to the species delimitation for prokaryotes. Stackebrandt and Goebel (1994) initially proposed a >97.5% similarity threshold for the 16S rRNA gene, later adjusted to 98.7%–99% by Stackebrandt and Ebers (2006). However, the 16S rRNA gene, which better serves generic delimitations, is too conserved to recognize closely related species (Dvořák, Jahodářová, et al., 2015, Dvořák, Poulíčková, et al., 2015, Dvořák et al., 2023 for details). Richter and Rosselló‐Móra (2009) proposed a 95%–96% average nucleotide identity (ANI; sequence similarity over the whole genome) as a standard for species delimitation. Later, Jain et al. (2018) advocated a 95% threshold based on a 90k genome data set. Moreover, Konstantinidis and Tiedje (2005) introduced the concept of the average amino acid identity (AAI) threshold, which uses amino acid sequences instead of the whole genome nucleotide sequence (AAI can be calculated using a pipeline developed by Kim et al., 2021). However, genetic similarity approaches share a key limitation: Similarity matrices fail to account for varying speciation rates across lineages. Some bacterial groups accumulate mutations faster than others, making the 95%–96% ANI threshold unsuitable for all groups (Stanojković et al., 2024; reviewed in Dvořák et al., 2023; Hugenholtz et al., 2016). For instance, data from 36,781 bacterial and archaeal species (including cyanobacteria) showed ANI values between 83% and 95% (Chuvochina et al., 2022). Although similarities may not reflect the complexity of speciation, they are still helpful for species identification and as an initial hint for species delimitation.

## 16S–23S ITS rRNA REGION SECONDARY STRUCTURES

The ITS rRNA region sequences are used in systematics because they are more variable (phylogenetically informative) than the 16S rRNA gene, and the RNA structures are frequently used for species designations (Boyer et al., 2001). The sequences of the most commonly employed helices used in comparative analyses within the 16S–23S ITS rRNA region (D1‐D1′, Box B, and V3) can be identified by a manual search of the ITS rRNA region sequence, or they can be identified using the Cyanobacterial ITS Motif Slicer (CIMS; Labrada et al., 2023; https://github.com/nlabrad/CIMS‐Cyanobacterial‐ITS‐motif‐slicer). Based on the identified sequences, the secondary structures are estimated using websites such as mfold (Zucker, 2003) and SPOT‐RNA (Singh et al., 2019). To obtain the secondary structure of the beginning of the ITS rRNA region (i.e., the D2 region), the sequence of the leader, which can be obtained from many genomes, is essential. To obtain the end of the ITS rRNA region (i.e., the V3 and D5 regions) secondary structure, we need to sequence the 23S–5S ITS rRNA region.

Alas, there are some caveats about using ITS rRNA regions for taxonomy purposes, so please read and be advised of a few previous studies focused on this (e.g., McGovern et al., 2023; Skoupý et al., 2024; Villanueva et al., 2024). When comparing folded structures from taxa with multiple operon copies, secondary structures originating from paralogs may potentially lead to phylogenetic errors. There is evidence that Box B and V3 helices are well homogenized between paralogs and can be compared. D1‐D1′ helices will vary among orthologous operons, but often the core sequence of D1‐D1′ helices in paralogs is considerably different (Bohunická et al., 2024; Saraf et al., 2024; Villanueva et al., 2024), which may lead to erroneous conclusions. Authors publishing secondary structure comparisons should take care to indicate not only the genus, species, and strain for helical structures, but also designated orthologs or paralogs if applicable. The ITS rRNA region orthologs or paralogs can be identified by the approach of Villanueva et al. (2024). Due to the mentioned difficulties, we recommend keeping the secondary structures only as an optional supporting criterion. Phylogenetic analysis and dissimilarity comparisons using ITS rRNA region sequence data are likely more informative than secondary structure comparisons, particularly when multiple operons are detected (Pietrasiak et al., 2019, 2021).

## SPECIES DELIMITATION AND FORMAL DESCRIPTION

### Species concept and definition

Ben Franklin once famously quipped: “in this world nothing can be said to be certain, except death and taxes.” We note one more thing for inclusion: the everlasting debate on species concepts and definitions. We will not discuss this issue here, since it has been reviewed elsewhere (e.g., Dvořák et al., 2023; Dvořák, Jahodářová, et al., 2015; Dvořák, Poulíčková, et al., 2015; Johansen & Casamatta, 2005; Kollár et al., 2022; Wilkins, 2022; Zachos, 2016), but by far the most commonly employed concept among cyanobacteriologists is the monophyletic species concept sensu Johansen and Casamatta (2005). This concept holds that a species is the lowest monophyletic unit diagnosable via an apomorphy that is worthy of recognition. It bears noting that these apomorphies may be morphological, ecological, genetic (e.g., inserts or unique sequences, ITS rRNA region patterns), molecular (e.g., the presence of a compound only certain taxa can produce), etc. Although it is widely applied, it is only rarely mentioned explicitly. Alternatively, with the availability of the population genomic data for the studied genera, a modified biological species concept could potentially be applied. This concept identifies barriers to gene flow between the species; gene flow is more frequent within the species than between the species (Dvořák et al., 2023). *We recommend an explicit statement on the species concept applied to each proposed taxon; this would facilitate reproducibility of the taxonomic reasoning.*

### Codes of nomenclature

Cyanobacterial taxa can be named under three codes: the International Code of Nomenclature of Algae, Fungi, and Plants (ICN; https://www.iaptglobal.org/madrid‐code‐online), the International Code of Nomenclature of Prokaryotes (ICNP; https://www.the‐icsp.org/index.php/code‐of‐nomenclatur), and the Code of Nomenclature of Prokaryotes Described from Sequence Data (SeqCode; https://www.isme‐microbes.org/seqcode‐initiative; Hedlund et al., 2022). These codes differ from one another with regard to what each one does or does not require for the named taxon to be considered valid, and each accommodates different nomenclatural types.

The ICN, also known as the “botanical code,” traditionally included (and still does include) cyanobacteria. This code requires metabolically *inactive* samples (e.g., herbarium specimens from the natural habitat, dried strains, chemically preserved samples, etc.) as a type, an illustration in the main document (i.e., not as a supplemental figure), and a formal description in a journal or book (see ICN for the specific requirements). These types need to be deposited in collections readily available to the scientific community, preferentially at least two sites. Neither a strain nor culture is required for description under the ICN, allowing for descriptions of uncultured taxa. However, we recommend designation of reference cultured strains for the taxon proposal under ICN if possible.

The ICNP requires that two axenic cultures be deposited in culture collections in two different countries as a type and description of the taxa in the International Journal of Systematic and Evolutionary Microbiology (IJSEM) or (if published outside of IJSEM) validation by the IJSEM (https://www.microbiologyresearch.org/content/journal/ijsem). Given the difficulties in obtaining axenic strains, the ICNP is not often employed for cyanobacteria (and, in fact, precludes validation). It is also problematic because some countries have laws preventing researchers from depositing cultures outside of the country as well as preventing cultures from being subsequently mailed outside of the country (which invalidates the taxon).

The SeqCode enables a proposal of a new taxon using only a high‐quality genome sequence as type material. These sequences may be obtained from isolates, from single cells, or from metagenomes. Strains and morphology are not required, nor are 16S rRNA gene sequences. Although the employment of SeqCode has some notable advantages (e.g., high‐quality whole genome sequence, flexibility of employing axenic strains or not), it does have some drawbacks (in the context of cyanobacterial taxonomy specifically) that may be difficult to overcome. Without any sort of anchoring to a physical sample, potentially valuable information may be lost or never evidenced. Characters such as environmental or culture‐induced plasticity, life‐cycle features, etc., would be potentially missed. Further, without any sort of physical sample, future analyses on the material would not be possible, making ties to historic samples tenuous.

Although the International Committee on Systematics of Prokaryotes (ICSP) has not accepted the SeqCode at present, there is an unusual path by which names published under the SeqCode are valid regardless. Article 45.1 of the ICN states “If a taxon originally assigned to a group not covered by this *Code* is treated as belonging to the algae or fungi, any of its names need satisfy only the requirements of the relevant other *Code* that the author was using for status equivalent to valid publication under this *Code…*”; this rather clearly states that bacterial taxa validly described under the SeqCode are valid under the ICN if considered to be algae, which they are. The ICNP was recently emended to have reciprocity with the ICN so that taxa of cyanobacteria valid under the ICN are also valid under the ICNP (Oren et al., 2023). Consequently, an argument could be made that if a cyanobacterial taxon is valid under the SeqCode, it is valid under the ICN, and is consequently valid under the ICNP, even though the ICSP rejects the SeqCode. However, it must be noted that proposals to recognize new taxa based solely on sequences were *not* included in the recently published Madrid Code (Turland et al., 2025). *Thus, we strongly recommend that researchers do not use the SeqCode until when, and if, it gains wider acceptance, possibly through revision of the code to solve the aforementioned issues. At this time, we maintain that the ICN be the main code used for Cyanobacteria. In any case, it is crucial to include a reference to the Code you use for any taxonomic proposal.*

### Specimens and strains

Depending on the code employed, nomenclatural type materials must be submitted to a collection (see above). We strongly recommend that the nomenclatural type material be stored in a curated collection, which has a history of loaning materials and not in someone's personal collection. We also encourage the deposition of isotypes (duplicates) where possible. When depositing, there should be enough material for several DNA extractions and morphological observation so that the specimen is not completely destroyed upon reexamination. Furthermore, we recommend short‐read sequencing, because herbarium specimen DNA quickly disintegrates into shorter fragments. The PCR with subsequent 16S rRNA gene sequencing tends to produce short fragments of several hundred bases (Dvořák et al., 2020), which have only a limited taxonomic resolution (reviewed in Dvořák, Jahodářová, et al., 2015; Dvořák, Poulíčková, et al., 2015; Dvořák et al., 2023). Unique accession numbers (e.g., “barcodes” provided by the collections that identify the deposited strain) *must* be stated in the taxon proposal. If the herbarium specimen is selected as the nomenclatural type, make sure that enough biomass of the sample is stored. We recommend dried, monoclonal cultures in an envelope or on paper as the herbarium specimens because the natural samples may contain many species of cyanobacteria. Herbarium specimens have been sequenced after 100 (Dvořák et al., 2020) and 200 years (Skoupý et al., 2024; Stanojković et al., 2024), whereas formaldehyde‐ or alcohol‐fixed samples may be difficult to sequence using regular approaches (Hykin et al., 2015). The list of available herbarium collections (see Index Herbariorum: https://sweetgum.nybg.org/science/ih/; Thiers, 2025) and the database of strains in culture collections can be found here: https://straininfo.dsmz.de/.

### Data management and reproducibility

Taxonomic research has been producing vast amounts of data connected to strain biology and evolution. The most common forms of data in taxonomy are morphological measurements, environmental data, and the actual DNA sequences (including folded ITS rRNA region structures). Further, many studies provide data about chemical compounds produced by cyanobacteria such as secondary metabolites. As we stressed in the previous section, freely available information about the species is crucial for the reproducibility of the data in taxonomy (e.g., chemotaxonomy).

All DNA sequences should be stored in NCBI GenBank (https://www.ncbi.nlm.nih.gov/), which is the most comprehensive and regularly updated database containing both curated sequences and a raw sequencing data archive. Note that the sequences can also be submitted to the European Nucleotide Archive (ENA; https://www.ebi.ac.uk/ena/browser/home) and the DNA Data Bank of Japan (DDBJ; https://www.ddbj.nig.ac.jp/index‐e.html). All three databases have regular data exchange, and therefore, the data need only be submitted to one of them (see https://www.ncbi.nlm.nih.gov/genbank/submit/). Metagenomes, single‐cell assembled genomes, and genomes of cultured strains can be submitted to GenBank as assemblies or as raw data. The raw data should be submitted to the Sequence Read Archive (https://www.ncbi.nlm.nih.gov/sra), also powered by NCBI.

Multiple sequence alignments (MSAs) for each tree should be stored in a public repository such as Dryad (https://datadryad.org/stash) or figshare (https://figshare.com/), along with the tree files in newick or nexus formats. These repositories can also be used to store any other data related to the studied material (e.g., tables of cell dimensions, photographs, and videos). Most journals now allow supplemental material including these types of data as well.

One crucial issue in cyanobacterial taxonomy is the erroneous taxonomic assignment of sequences in GenBank. If researchers are less than diligent, out‐of‐date (or just inaccurate) taxonomic assignments may lead to flawed taxonomic assumptions (e.g., strains labeled as “*Leptolyngbya*” are rampant in GenBank, yet only a handful of these strains represent the actual *Leptolyngbya* sensu stricto). Dvořák et al. (2018) estimated that ~80% of the cyanobacterial species names of 16S rRNA gene sequences are flawed. The primary reason for this lies in the fact that researchers rarely update taxon names associated with sequences deposited in GenBank (Turner, 2019). This problem is exemplified by the use of “collector taxa” names such as “*Phormidum* sp.” The genus *Phormidum* was originally thought of as “cosmopolitan” and is both environmentally plastic and cryptic, meaning that many researchers isolating simple, character‐poor filaments assumed they had isolated “*Phormidium*,” as there was nothing particularly noteworthy in the morphology. These strains made their way into papers and culture collections, leading to taxonomic pollution (i.e., a commonly misidentified strain later becomes sequenced, leading later researchers utilizing that strain sequence to continue using “*Phormidium*”). The curated CyanoSeq database (Lefler et al., 2023), in which the taxonomic identifications of 16S rRNA gene sequences are updated, provides a solution to this issue by producing a more robust, accurate taxonomy, although it does not address sequences and strains already in GenBank. The authors should consider submitting their sequences to this database to keep it as complete as possible.

### Species distribution

A sizable percentage of new taxa described over the last decade represent novel, monospecific genera. Although this may suggest that these species are endemic to their respective regions, they are often soon observed at different locations. For example, *Brasilonema* (Fiore et al., 2007) was initially described from the bromeliads in Brazil and later observed in a myriad of other habitats and locations (i.e., Barbosa et al., 2021; Bohunická et al., 2024; Villanueva et al., 2018). With a wealth of sequence data in the Sequence Read Archive of NCBI and GenBank, it is now possible to screen for the distribution of a new taxon immediately upon the taxon proposal. For instance, we observed a new genus of the filamentous cyanobacterium *Argonema* in the biological soil crusts of Antarctica (Skoupý et al., 2022). However, *Argonema* may be an overlooked genus distributed in the biological soil crusts from the tropics to both poles, thus cosmopolitan (Skoupý et al., 2022). Another example is *Komarekiella*: Originally observed on a concrete wall in the Brazilian Atlantic rainforest and in puddles in a concrete patio in Hawaii (Hentschke et al., 2017), it has subsequently been observed in very disparate environments, such as within European lichens and colonizing dolphin skins (Brown et al., 2021; Jung et al., 2021). We suggest that the distributional data could be part of each taxonomic study.

### Formal descriptions

The key part of the taxonomic proposal is the formal description. As most proposals are made under the ICN, please consult the ICN rules of nomenclature (https://www.iaptglobal.org/madrid‐code‐online). Almost all mistakes that researchers make when using the ICN are due to improper designation of the holotype. Holotypes cannot be living cultures (unless immobilized in a metabolically inactive form, such as via cryopreservation, drying, stubs for electron microscopy, etc.) and, instead, must be a single designated specimen.

Orthographic mistakes are also common, although they do not invalidate the new name. To prevent orthographic errors, we recommend researchers utilize Brown (1956), Stearn (2004), and Turland et al. (2025). It is also *essential* to check whether the name you have chosen has been used before (for the ICN, this can be found on the International Plant Names Index: https://www.ipni.org/). Since fungal generic names are not included in IPNI, the *Index Fungorum* (https://www.indexfungorum.org/names/names.asp) should also be consulted for fungal names. *Extensive* and *critical* guidelines for proper taxonomic descriptions are outlined in three articles in *Notulae Algarum*: Nomenclatural FAQs (https://www.notulaealgarum.com/nomenclature/index.html), Validating a Species Name (https://www.notulaealgarum.com/nomenclature/is_my_name_valid.html), and Honorific Epithets (https://www.notulaealgarum.com/nomenclature/personal_epithet_formation.html). Any researcher describing new taxa for the first time (or even for the 10th time) should make use of these clearly explained guides. Always make sure that the new name has not been used for another organism in the respective Code. Reviewers would do well to review the guides as well when reviewing taxonomic manuscripts.

We summarize essential components of a valid taxon description below. We attempted to provide guidelines as complete as possible. For examples of valid taxon descriptions, see Kaštovský et al. (2014), Jung et al. (2020), Barbosa et al. (2021), Berthold et al. (2021), Johansen et al. (2021), McGovern et al. (2023), and Dvořák et al. (2024). We encourage editors and reviewers to reject papers that do not deposit taxa in collections recognized in the Index Herbariorum (see **Holotype** below).

### Components of valid species proposal under the ICN

**Class:** *Cyanophyceae*

**Order:** “*Lorem ipsum*” Authority

**Family:** “*Lorem ipsum*” Authority

*Genus species* Authority sp. nov.

**Figure reference:** References to the figures pertaining to the specimen material being described.

**Description:** See the Phenotype analyses section above for details. Generally, descriptors are introduced in an organized specific manner from macroscopic to microscopic, or larger features to smaller ones; that is, thallus, filaments, sheath, trichomes, cells, and ultimately reproduction.

**Diagnosis:** Although not strictly required per se, a diagnosis (e.g., how is this taxon different or distinguishable from others, in particular sister species) can be very helpful. This might be a morphological feature (e.g., a unique phenotype), an interesting ecology (e.g., only observed in polar‐bear hairs), a genetic feature (either sequence divergence or, even better, an insert), a unique ITS rRNA region folding pattern, or any combinations thereof. Although helpful, diagnoses may be less useful than well‐articulated descriptions, since taxa described after the publication may necessitate differential diagnoses (e.g., a species described in the future may wind up being more closely related).

**Etymology:** “descriptor” (L., Latin or G., Greek). Select an appropriate name. Both Greek and Latin can sometimes be tricky for us folks in the modern era, so consulting with a colleague may be appropriate and fruitful. Also, remember that Latin and Greek words may not be what one anticipates from English. For example, if something is brown in coloration, you would not use *brownii* (this would be naming the taxon after someone named Brown); instead, it would be *fuscus*. Lastly, remember that one cannot name taxa after themselves (otherwise, the world would be rife with *casamattae*). We refer the reader to (https://www.notulaealgarum.com/nomenclature/index.html) for more information.

**Holotype:** Herbarium acronym (as cited in the Index Herbariorum, https://sweetgum.nybg.org/science/ih/); HERBARIUM ACCESSION # (Statement of preservation; i.e., dried material in a metabolically inactive state of reference strain XXXX), deposited in XXXXX. Include as much information about the location as possible (e.g., Country, State, City, Location, GPS). Should be as specific as possible, including GPS if available. GPS can help you provide more information from the environmental databases about the investigated locality, which includes temperature, precipitation, radiation, soil properties, etc. (see Skoupý et al., 2024; Stanojković et al., 2024). Fresh/marine/terrestrial/epibiont and ecosystem classification (https://gold.jgi.doe.gov/ecosystem_classification). Collector information is also useful: Consider the collector, collection number, date of collection, habitat, etc.; anything that might facilitate future employment of the specimens.

**Reference strain:** If the description is based on the cultured strains, only one strain per taxon is submitted to a recognized culture collection, so it is available to other researchers. Note that under the ICN, the strains do not have to be axenic.

**Habitat:** Where can the taxon be located? Is it planktonic? Benthic? Epilithic? Additionally, in what environmental context? Marine? Freshwater? Soil crusts? Refer to Sampling and Strain Isolation section for details.

**Reference sequences:** Statement of source 16S rRNA gene, 16S‐23S ITS rRNA region, and genome with accession number in GenBank or other relevant database. The sequence should preferably be obtained from the reference strain. However, for uncultured species, sequence from other materials is also possible (single cell/filament/colony; see DNA Sequencing section).

**Materials analyzed:** List any or all strains analyzed to erect this specific entity.

### Components of valid higher taxa proposal under the ICN

**Class**: *Cyanophyceae*

*Genus, family, order*, etc. Authority gen. nov., fam. nov., ordo nov. etc.

**Description:** For novel genus erection, it is good practice to begin with the basic morphological description such as either homocytous or heterocytous, multiseriate/uniseriate, isopolar/homopolar, branching/non‐branching, cyanobacteria. See Phenotype Analyses section for some features to articulate. Generally, descriptors are introduced in an organized specific manner from macroscopic to microscopic, or larger features to smaller ones, that is, thallus, filaments, sheath, trichomes, cells, and ultimately reproduction. For the taxa higher than genus, the description would be more general.

**Type taxon** (type species, genus, family, etc.) Authority

**Etymology:** See above.

### Additional nomenclatural terminology

Alas, the terminology behind these endeavors can be rather confusing. Thus, we provide some assistance with some of the more common, yet potentially arcane, terms:

*Basionym*: Previously published legitimate synonym from which a new name is formed for a taxon; for example, the basionym for *Limnothrix redekei* (van Goor) Meffert (1988) is *Oscillatoria redekei* van Goor (1918).

*Epitype*: Specimen or illustration that serves as additional type material when the holotype or lectotype of a validly published taxon is demonstrably ambiguous and cannot be critically identified for the purpose of the precise application of the name to a taxon. Note that in order to designate an epitype, it is required to cite the holotype or lectotype, and it is good practice to say why the holotype or lectotype is ambiguous or missing. If the holotype or lectotype is missing, then a neotype must be designated (note: illustrations that serve as types from the distant past are no longer allowed).

*Holotype*: The sample or image designated as the nomenclatural type. It must be a single specimen or gathering (e.g., a sample consisting predominantly of the organism in question, but perhaps with other concurrent taxa).

*Iconotype*: An illustration that served as the basis for describing a new species, *no longer allowed by the present ICN*.

*Isotype*: A duplicate specimen of the holotype.

*Lectotype*: Sample or image designated from the original material as the nomenclatural type if the holotype was not indicated at the time of publication. Another reason for designating a lectotype may include the destruction of the original type (e.g., after the destruction of the Berlin Herbarium).

*Neotype*: A specimen or illustration selected to serve as a nomenclatural type if no original material is extant or as long as it is missing.

*Paratype*: Any specimen cited in the protolog that is neither the holotype nor an isotype, nor one of the syntypes if in the protolog two or more specimens were simultaneously designated as types.

*Nomenclatural type*: The element to which the name of a taxon is permanently attached.

## CONCLUSIONS

Articulating cyanobacterial diversity is both challenging and rewarding. These organisms are exceedingly significant both ecologically and evolutionarily, and proper identification can be of paramount importance for management, industry, food technology, etc. Long neglected by both microbiologists and botanists, the cyanobacteria are experiencing something of a renaissance in terms of investigators attempting to explore their biodiversity. New technologies have greatly increased our ability to explore the genetic divergence, biogeography, and basic biodiversity of this enigmatic lineage. We have hopefully provided step‐by‐step guidelines for good practice in the taxonomy of Cyanobacteria. We acknowledge that we may not be able to find one solution to every taxonomic question that may be posed. However, we would like to motivate taxonomic novices, established researchers, and reviewers to reflect on these presented guidelines to improve taxonomic practice in our beloved group of organisms.

## AUTHOR CONTRIBUTIONS

**Petr Dvořák:** Conceptualization (equal); writing – original draft (equal); writing – review and editing (equal). **Svatopluk Skoupý:** Conceptualization (equal); writing – original draft (equal); writing – review and editing (equal). **Aleksandar Stanojković:** Writing – original draft (equal); writing – review and editing (equal). **Jeffrey R. Johansen:** Writing – original draft (equal); writing – review and editing (equal). **Chelsea Villanueva:** Writing – original draft (equal); writing – review and editing (equal). **Patrick Jung:** Writing – original draft (equal); writing – review and editing (equal). **Laura Briegel‐Williams:** Writing – original draft (equal); writing – review and editing (equal). **H. Dail Laughinghouse IV:** Conceptualization (equal); writing – original draft (equal); writing – review and editing (equal). **Forrest W. Lefler:** Conceptualization (equal); writing – original draft (equal); writing – review and editing (equal). **David E. Berthold:** Conceptualization (equal); writing – original draft (equal); writing – review and editing (equal). **Jan Kaštovský:** Writing – original draft (equal); writing – review and editing (equal). **Anne C. Hurley:** Conceptualization (equal); writing – original draft (equal); writing – review and editing (equal). **Dale A. Casamatta:** Conceptualization (equal); writing – original draft (equal); writing – review and editing (equal).
